# The *Vibrio splendidus* Ferric Uptake Regulator Promotes Infection of *Apostichopus japonicus* Through Activation of the Type VI Secretion System

**DOI:** 10.3390/microorganisms14071508

**Published:** 2026-07-10

**Authors:** Jiqing Liu, Xinyue Ma, Lincheng Hu, Zhimeng Lv, Yina Shao, Chenghua Li, Weikang Liang

**Affiliations:** 1 State Key Laboratory of Agricultural Products Safety, Ningbo University, Ningbo 315211, China; liujiqing@nbu.edu.cn (J.L.); 15245898394@163.com (X.M.); hlc282503@163.com (L.H.); lvzhimeng@nbu.edu.cn (Z.L.); shaoyina@nbu.edu.cn (Y.S.); 2 Laboratory for Marine Fisheries Science and Food Production Processes, Qingdao Marine Science and Technology Center, Qingdao 266237, China

**Keywords:** ferric uptake regulator, *Vibrio splendidus*, aggregation, adhesion, type VI secretion system

## Abstract

Iron is essential for bacterial survival and pathogenicity. During infection, bacteria face iron limitation imposed by the host and must develop strategies to obtain iron and maintain virulence. The ferric uptake regulator (Fur) is a major iron-responsive regulator that coordinates iron metabolism and virulence-related functions in many bacteria. However, whether and how Fur regulates virulence systems in the *Apostichopus japonicus* pathogen *Vibrio splendidus* remains unclear. In this study, the *VsFur*-knockdown strain was used to investigate the role of *VsFur* in T6SS regulation and virulence-associated traits. Aggregation and adhesion assays showed that knockdown of *VsFur* reduced bacterial aggregation and adhesion to coelomocytes of the *A. japonicus*. qPCR and Western blot analyses revealed that iron limitation and *VsFur* knockdown both significantly inhibited the expression of T6SS-related genes and the secretion of Hcp. In terms of mechanism, VsFur directly bound to the promoter region of the T6SS gene cluster and positively regulated its transcription. ELISA and immunofluorescence analyses further showed that knockdown of *VsFur* significantly reduced the secretion and host–cell delivery of the T6SS effector proteins Hcp and VgrG. Moreover, hemolysis and siderophore production assays showed that *VsFur* knockdown enhanced hemolytic activity and siderophore production, suggesting the existence of a compensatory mechanism. Collectively, these results reveal a novel regulatory relationship between *VsFur* and virulence in *V. splendidus*, providing new insights into iron-dependent virulence regulation and a theoretical basis for the development of novel strategies for the prevention of bacterial diseases in *A. japonicus* aquaculture.

## 1. Introduction

*Vibrio splendidus* is a group of Gram-negative bacteria widely distributed in the marine environment, and its pathogenic mechanism involves multiple strategies, such as adhesion, invasion, toxin secretion, and iron competition [1]. As an example, *V. splendidus* is the main pathogen of the *Apostichopus japonicus* rotting skin syndrome, and its pathogenic process is highly dependent on the acquisition of iron ions [2]. It has been shown that *V. splendidus*, through the secretion of isohydroxamate-type iron carriers to complex environmental Fe^3+^, which is taken up via the IutA receptor-mediated transporter system, or via the Hppd enzyme-catalyzed formation of HGA-melanin to reduce Fe^3+^ to Fe^2+^, which is then taken up via the FeoAB transporter system [3], forms a dual-pathway iron uptake network. Iron is not only an essential cofactor for bacterial growth but also regulates the expression of virulence factors such as hemolysin (4-HPPD) and metalloproteinases (Vsm). For example, a low-iron environment significantly induced the expression of the *V. splendidus VsFur* gene, suggesting the pivotal position of the iron uptake regulator (Fur) in virulence regulation [4].

Ferric uptake regulator (Fur) is a core regulatory protein of bacterial iron metabolism, and its function is highly conserved across a wide range of pathogens [5]. Fur represses the transcription of iron uptake-related genes by dimerization of the promoter region of target genes with Fe^2+^ as a cofactor, whereas in iron deficiency, Fur monomers dissociate to activate the iron carrier synthesis and transport system [6]. For example, knockdown of the Fur gene in *Riemerella anatipestifer* resulted in an 80-fold reduction in virulence and a significant down-regulation of the expression of genes involved in iron carrier synthesis [7]. A similar mechanism was demonstrated in *Pseudomonas aeruginosa*, where Fur not only regulates iron homeostasis but also affects periplasmic membrane formation and resistance to oxidative stress [8]. Studies have revealed that Fur functions as a global regulator that dynamically balances iron metabolism and virulence expression through promoter binding [9].

As a “molecular switch” for gene transcription, promoters determine the spatial and temporal specificity of gene expression through their sequence features and interactions with regulatory proteins [10]. Fur precisely regulates iron metabolism genes by recognizing the conserved sequence Fur-box in the promoter region [11]. It has been shown that in *Salmonella,* mutations in the Fur-binding site can lead to constitutive expression of iron carrier synthesis genes and exacerbate intra-host iron competition [12]. In addition, the application of synthetic promoter technology has further validated the decisive influence of promoter structure on the intensity of gene expression. For example, the activity of the iron carrier secretion system of *P. aeruginosa* can be targeted to enhance its environmental adaptability by designing specific promoters [13]. These cases emphasize the centrality of promoter-binding mechanisms in the evolution of bacterial adaptations.

The T6SS is a widespread nanoinjection device in Gram-negative bacteria that mediates cytotoxicity, immune escape, and ecological niche competition by translocating effector proteins into host cells or competing bacteria [14]. *Vibrio cholerae* secretes the effector protein VgrG1 via the T6SS, which targets host cytoskeletal proteins (G-actin), inducing cellular damage and disruption of immune defenses [15]. Additionally, this secretion system enables *V. cholerae* to scavenge intestinal commensal bacteria and promote colonization dominance by secreting lysozyme-like proteins [16]. It is noteworthy that the expression of the T6SS is often regulated at multiple levels by environmental signals and transcription factors. For example, Fur in *Aeromonas hydrophila* directly activates the transcription of the T6SS gene cluster, and knockdown of Fur leads to defective T6SS assembly and a significant decrease in virulence, which provides a paradigm for studying the functional coupling of iron metabolism and secretion systems [17].

Recent studies have gradually revealed that there is a close interplay between iron homeostasis and T6SS function. On the one hand, T6SS may hijack host iron resources by secreting iron-chelating proteins [18]. On the other hand, iron signaling may directly regulate the synthesis and secretion of T6SS effector proteins through regulatory factors such as Fur [19]. In *V. splendidus*, although the immunosuppressive function of T6SS and the diversity of the iron uptake pathways have been clarified, whether Fur regulates the expression of the T6SS gene promoter by binding to it is still unknown. Dissecting this regulatory network will not only elucidate the role of the “iron-T6SS” axis in pathogen adaptation and virulence evolution, but also provide a theoretical basis for the development of novel antimicrobial strategies targeting the cross-cutting pathways of the iron-metabolism-secreting system [20]. For example, small-molecule inhibitors targeting Fur-T6SS axis may block both bacterial iron uptake and virulence, offering dual protection to the host.

Our previous transcriptomic analysis showed that knockdown of VsFur altered the expression of multiple genes associated with motility and secretion systems in the *A. japonicus* pathogen *V. splendidus* [21]. Based on these findings, we intend to systematically analyze the transcriptional regulatory network of Fur protein on the T6SS gene cluster, thereby elucidating how iron signaling drives virulence effects through molecular switching mechanisms and providing new targets for aquaculture disease prevention and control. This study will fill the theoretical gap in the cross-regulation of iron metabolism and secretion system in Vibrio and promote the cross-innovation of pathogen biology and synthetic regulatory technology.

## 2. Materials and Methods

### 2.1. Bacterial Strains and Plasmids

*V. splendidus* strain was isolated from the chemodermal tissues of the *A. japonicus* suffering from “rotting skin syndrome” and preserved in our laboratory, and the strain was deposited in the Chinese Microbial Strain Collection Center under CGMCC No. 7.242. *Escherichia coli* DH5α receptor cells and *E. coli* BL21(DE3) receptor cells were purchased from Takara (Shiga, Japan). The vectors used in this experiment were mainly pMD19-T (Takara, Shiga, Japan), pET-28a (stored), and pSC11 (stored). The bacterial strain was stored at −80 °C in 50% (*v*/*v*) glycerol until use.

### 2.2. Western Blot Analysis

Protein lysate containing loading buffer was separated by SDS-PAGE. After SDS-PAGE, the proteins were transferred to PVDF (Merck, Darmstadt, Germany) membranes by the transmembrane assay. Then, the membranes were incubated with TBST containing 5% skimmed milk powder at room temperature for 2 h. After that, the membranes were incubated with polyclonal antibody (diluted 1:1000 in TBST with 10% BSA) overnight at 4 °C. The membranes were then washed with TBST. After washing three times with TBST for 10 min, they were incubated with goat anti-mouse or goat anti-rabbit IgG (diluted 1:2000 in TBST with 10% BSA, Beyotime, Shanghai, China) for 2 h at room temperature. After washing three times with TBST, the membranes were incubated with Western Lightning-ECL substrate (NCM Biotech, Suzhou, China), and the chemiluminescent signals were visualized using X-OMAT AR X-ray film (Bio-Rad, Hercules, CA, USA).

### 2.3. Quantitative Real-Time PCR Analysis

Total RNA was extracted from coelomocyte samples from different groups using TRIzol reagent. Then, 1 μg RNA was reverse-transcribed to cDNA using a PrimeScript RT Kit with gDNA Eraser (TaKaRa, Shiga, Japan). The relative mRNA expression of each gene was analyzed using an Applied Biosystem 7500 Real-time Quantitative PCR System (Thermo Fisher Scientific, Waltham, MA, USA) and TB Green^®^ Fast qPCR Mix (TaKaRa, Shiga, Japan). Each reaction contained a volume of 20 µL, which included 8 µL cDNA (1 ng/µL), 0.8 µL each primer (10 µM), 0.4 µL ROXII, and 10 µL TB Green PCR Master Mix (TaKaRa, Shiga, Japan). The amplification procedure was as follows: denaturation at 95 °C for 5 min, followed by 40 cycles of 95 °C for 5 s and 60 °C for 34 s. After the cycling stage, melting curve analyses were performed. Each group trial was run in triplicate, with parallel reactions and repeated thrice. The primer sequences used for qRT-PCR are listed in Table 1. For normalization purposes, *A. japonicus* β-actin (Ajβ-actin) served as the internal reference gene. Relative expression levels of target genes were calculated using the 2^−ΔΔCT^ method and are presented as fold changes relative to the control group. Data are expressed as the mean ± SD from three independent experiments (*n* = 3).

### 2.4. Prediction of Promoter Regions

The promoter functional region of the Fur-binding cassette-containing DNA fragment of the screened *V. splendidus* T6SS gene cluster was predicted using the promoter online prediction website SoftBerry (http://www.softberry.com). The promoter functional regions of the DNA fragments were analyzed by selecting and entering the Annotation of Bacterial Genomes module on the SoftBerry website, and then selecting the BPROM Bacterial Promoter Analysis function [22].

### 2.5. Promoter Activity Assay

The predicted promoter fragments were amplified using design-specific primers (Table 1), and the promoter activity assay recombinant plasmid was constructed with the digested pSC11 vector and transformed into *E. coli* DH5a sensory state. After that, the promoter activity was detected using the X-gal assay and the ONPG assay, respectively.

For the X-gal assay, the constructed strain was shaken in a shaker at 28 °C until OD600 = 0.4–0.6; 100 μL of the constructed strain was evenly coated on the solid medium containing X-gal (Sangon Biotech, Shanghai, China), and then cultured overnight in an incubator at 28 °C. The strength of the promoter activity was determined according to the number of colonies and the blue color of the colonies.

For the ONPG assay, the bacterial solution was incubated until the OD600 was about 1.0, and then taken out and put on ice. A volume of 500 μL of bacterial solution was aspirated into a 1.5 mL centrifuge tube, and 12 μL of chloroform and 3 μL of 20% SDS were added, then shaken vigorously for 10 s. A volume of 30 μL of the cell lysate was added to 500 μL of the Z buffer, and 13 μL of the ONPG with a concentration of 4 mg/mL was added to the centrifuge tube, mixed well, and then incubated at 37 °C for 10 min. ONPG at a concentration of 4 mg/mL was added to the centrifuge tube, and the reaction was started at 37 °C in a water bath. The time for the solution to turn yellow was recorded. After the color changed to yellow, 500 μL of 1 mol/L Na_2_CO_3_ was added to abort the reaction, and the absorbance at 420 nm and 550 nm was measured. Z buffer (50 mL) contained 60 mM Na_2_HPO_4_, 40 mM NaH_2_PO_4_, 1 mM KCl, 0.1 mM MgSO_4_, 5 mM β-mercaptoethanol; pH = 7.0.

The enzyme activity units were calculated as follows: Miller Units = 1000 × (OD420 − 1.75 × OD550)/(T × V × OD600), where OD420 and OD550 are the readings of the reaction solution after the color development. OD600 is the bacterial density of the culture solution used for the color development analysis. T is the reaction time of the color development (min). V is the volume of the culture solution used for the color development analysis (mL). T is the color reaction time (min). Based on the data obtained from three independent replicate experiments, GraphPad Prism 9 software (GraphPad Software, San Diego, CA, USA) was utilized for graphical presentation [23].

### 2.6. EMSA Experiment

A double-stranded DNA probe containing the binding site of the target protein, with one of the strands modified with biotin at the 5′ end, was first designed and synthesized, annealed and quantified, and then set aside. Active purified protein and biotin-labeled probe DNA fragments were added to the reaction system and incubated for 20 min at room temperature to form protein DNA complexes. Subsequently, the samples were mixed with the uploading buffer, loaded onto a pre-cooled 6% nondenaturing polyacrylamide gel (0.5 × TBE buffer), and electrophoresed at 100 V for 40 min at 4 °C to separate the complex from the free probe. At the end of electrophoresis, the gel was transferred to a nylon membrane by the wet-transfer method (380 mA constant current, 30 min) and closed with 5% skim milk powder/TBST for 1 h to reduce nonspecific adsorption. Finally, the membrane was immersed in streptavidin-HRP (1:3000 diluted in 2% BSA/TBST, Sangon Biotech, Shanghai, China) and incubated for 30 min, washed in TBST, and then ECL luminescent solution was added dropwise. The migrating bands were captured using a chemiluminescence imager with dynamically adjusted exposure times, and protein-binding activity was analyzed by the complex hysteresis bands [24].

### 2.7. Primary Cell Culture of A. japonicus

After *V. splendidus* infection or other specific treatment conditions, the body cavity fluid was collected by dissecting the mouth area of *A. japonicus* with sterilized scissors. The collected *A. japonicus* body cavity fluid was filtered with a 200-mesh sieve silk to break up tissues and debris, and isotonic anticoagulant (0.48 M NaCl, 0.019 M KCl, 0.02 M EGTA, 0.068 M Tri-HCl, pH 7.4) was added in an equal volume. The cells were mixed well with the filtered body cavity fluid and centrifuged at 4 °C 800 *g* for 10 min to collect the lower layer of cells; the cells were resuspended with 10 mL of isotonic buffer (0.53 M NaCl, 0.001 M EGTA, 0.01 M Tri-HCl, pH 7.6), and then centrifuged at 4 °C 800 *g* for 10 min, and the washings were repeated twice; the cells were washed with L-15 medium (100 μg/mL Gm. 0.39 M NaCl, 100 U/mL double antibody) to gently blow the resuspended luminal cells, counted by flow cytometry and added to cell culture plates, and incubated overnight at 16 °C in a cell culture incubator until the cells adhered to the wall.

### 2.8. Enzyme-Linked Immunosorbent Assay (ELISA)

The 96-well plates were blocked with PBST containing 5% BSA and 0.05% Tween-20 for 3 h at 37 °C. 100 μL of 1 × 10^6^ primary somatic cells of ginseng were added to each well, and three strains of experimental bacteria cultured to OD600 = 0.6 were added to each well at a ratio of MOI = 1 for 12 h. After three washes with PBST, each well was incubated with a dilution (1:1000 dilution) of the corresponding antibody for 1 h at 37 °C. After another three washes with PBST, the wells were incubated with goat anti-mouse IgG secondary antibody dilution (1:3000 dilution) at 37 °C for 1 h. The last three washes were followed by development of the color with TMB color development solution and termination of the reaction by addition of 50 μL of 1 M HCl; the absorbance of the developed color was read with a UV–visible spectrophotometer at 450 nm. The absorbance of the developed color was then read with a UV–visible spectrophotometer at 450 nm. The reaction was terminated by adding 50 μL of 1 M HCl, and the absorbance of the developed color was read at 450 nm with a UV–visible spectrophotometer [25].

### 2.9. Immunofluorescence

After overnight culture, the culture medium was removed, and the coelomocytes were washed three times with PBS for 5 min each. The coelomocytes were then fixed with 4% paraformaldehyde for 20 min at room temperature. After fixation, the coelomocytes were washed three times with PBS for 5 min each, and then permeabilized with 0.5% Triton X-100 for 20 min at room temperature. Next, coelomocytes were washed three times with PBS for 5 min each. The coelomocytes were subsequently blocked with an antibody blocking solution for 2 h at room temperature. The primary antibody dilution was prepared according to the ratio of 1:1000, added directly after the closure, incubated at 4 °C overnight, and washed with PBS 3 times, each time for 5 min. After the closure, the secondary antibody dilution (1:2000) was added and incubated at 37 °C for 2 h. The antibody was washed with PBS 3 times, each time for 5 min. Cell membrane dye Dil (Beyotime, Shanghai, China) was prepared according to the ratio of 1:5000, added to the cell culture plate, incubated for 20 min, and washed with PBS for 3 times, 5 min each time. DAPI (Beyotime, Shanghai, China) was prepared according to the ratio of 1:5000, added to the cell culture plate, incubated for 10 min, and washed with PBS for 3 times, 5 min each time. The stained crawlers were taken out with a needle and tweezers, and one drop of Antifade Mounting Medium (Beyotime, Shanghai, China) was added; then, the crawler was inverted and covered on the slide for laser confocal microscopy observation [26].

### 2.10. Bacterial Aggregation

Aggregation tests were performed using previously described methods [27]. Bacteria were resuspended with PBS to an OD600 of 0.6, and the 0 h absorbance value (OD600, 0) was determined. Absorbance value (OD600, t) determination continued after 24 h of standing at room temperature, and three replicate experiments were performed for each experimental group. The formula for percent aggregation was X = (OD600, 0 − OD600, t)/OD600, 0 × 100%.

### 2.11. Bacterial Adhesion

The cultured *V. splendidus* bacterial solution was centrifuged at 5000 *g*/min for 10 min to collect the bacteria. Then, the bacteria were diluted with 2216E medium to a final concentration of 10^7^ CFU/mL. Coelomocytes were collected from the coelomic cavity of *A. japonicus* and inoculated into six-well plates containing cell-climbing slides. After incubation for 3 h, the coelomocytes were washed three times with PBS, followed by the addition of 1 mL of the diluted bacterial suspension. The coelomocytes were then incubated for a total of 1 h. After the incubation was completed, the cells were rinsed sufficiently and fixed in 4% paraformaldehyde in a refrigerator at 4 °C for 0.5 h. Then, they were subjected to Gram staining and placed in the oil lens of the light microscope to observe the differences in bacterial adherence. The number of bacteria in the same field of view was counted with the number of cells, and the bacterial adhesion rate was the ratio of the number of adherent bacteria to the number of adherent cells.

### 2.12. Hemolytic Capacity Assay

Qualitative determination of bacterial hemolytic activity: Wild-type strain (AJ01) and *VsFur*-knockdown strain (ΔVsFur) were coated on a 2216E plate containing 5% sheep blood, and incubated at 28 °C for 20 h, and the hemolytic ring was observed.

Quantitative determination of supernatant hemolytic activity: The culture supernatants of the strains were filtered using a 0.22 μm sterile filter membrane (Merck, Darmstadt, Germany) to obtain sterile supernatants. The sheep blood erythrocyte suspension (Klamar, Shanghai, China) was divided into 500 μL/tube after sufficient washing and 20-fold dilution (5% by volume) with pre-cooled DMEM. An experimental gradient was set up, with the first group consisting of 500 μL of prepared erythrocyte suspension with 100 μL of 2216E culture medium, the second group consisting of 500 μL of prepared erythrocyte suspension with 10 μL of strain supernatant and 90 μL of 2216E culture medium, and the third group consisting of 500 μL of prepared erythrocyte suspension with 50 μL of strain supernatant and 50 μL of 2216E culture medium. The third group was 500 μL of prepared erythrocyte suspension with 50 μL of strain supernatant and 50 μL of 2216E culture medium; the fourth group consisted of 500 μL of prepared erythrocyte suspension with 100 μL of strain supernatant. Each group was repeated three times. After incubation at 28 °C for 6 h, the sample was gently blown 10 times to release the heme, centrifuged at 10,000 *g* for 1 min at 26 °C, and the supernatant was taken to determine the OD570 value (the optimal absorbance wavelength of heme).

### 2.13. Iron Carrier Secretion Assay

Single colonies of AJ01 and ΔVsFur were inoculated into 2216E containing benzylpenicillin (Sangon Biotech, Shanghai, China) and incubated at 28 °C, 200 rpm overnight. The two strains were incubated until the OD600 was 1.0, and 5 μL of each strain was spotted on the same CAS plate, and then placed upside down in an incubator at 37 °C for 48 h. The iron carrier secreted by the strains combined with Fe^3+^ to cause an orange halo around the colonies, and the amount of iron carrier produced could be determined according to the size of the halo [28].

### 2.14. Statistical Analysis

All statistical analyses were performed using GraphPad Prism 9 (GraphPad Software, San Diego, CA, USA). Data are presented as the mean ± standard deviation (SD). Comparisons between two groups were performed using a two-tailed unpaired Student’s *t*-test, whereas comparisons among three or more groups were analyzed using one-way analysis of variance (ANOVA). In all cases, significance levels were defined as * *p* < 0.05, ** *p* < 0.01.

## 3. Results

### 3.1. The Knockdown of VsFur Affects Bacterial Aggregation and Adhesion Ability

**Figure 1:**
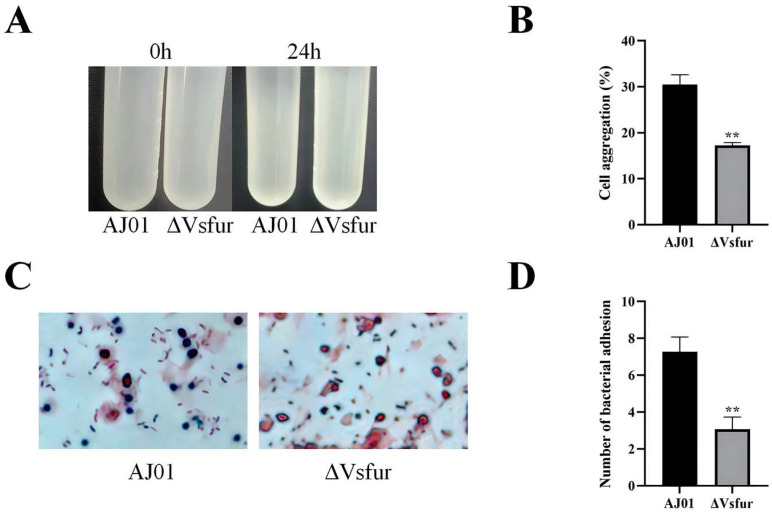
(**A**) Bacterial aggregation capacity assay of the two experimental strains, AJ01 and ΔVsFur. (**B**) Percentage of bacterial aggregation of the two experimental strains, AJ01 and ΔVsFur. (**C**) Adhesion capacity assay of the two experimental strains, AJ01 and ΔVsFur. (**D**) Average number of adherent bacteria of the two experimental strains, AJ01 and ΔVsFur. The error line is the standard deviation (*n* = 3). The asterisks represent significant differences (** *p* < 0.01 by two-tailed unpaired Student’s *t*-test).

Our previous transcriptomic analysis revealed that knockdown of *VsFur* significantly down-regulated genes involved in swarming motility, adhesion, and secretion pathways in *V. splendidus* [21]. To determine whether these transcriptional changes were accompanied by alterations in virulence-associated phenotypes, bacterial aggregation and adhesion abilities were evaluated. The results showed that knockdown of *VsFur* significantly impaired bacterial aggregation, resulting in a reduction of more than 13% compared with the wild-type strain (Figure 1A). In addition, the adhesion capacity of the mutant strain to *A. japonicus* coelomocytes was markedly decreased. The average number of adherent bacteria decreased by approximately 50% relative to the wild type (Figure 1B). Given the important roles of aggregation and adhesion in bacterial colonization and infection, these findings indicate that *VsFur* positively regulates virulence-related traits in *V. splendidus*.

### 3.2. VsFur Is Involved in the Regulation of T6SS in V. splendidus

**Figure 2:**
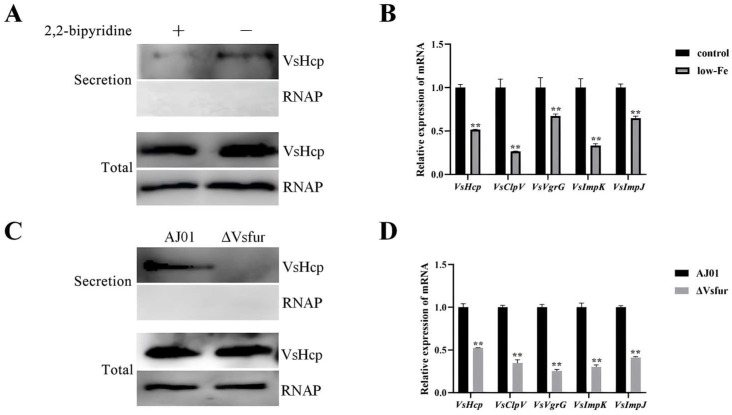
(**A**) Differences in VsHcp protein secretion of T6SS under low-iron and normal environmental conditions. (**B**) The mRNA expression levels of T6SS genes under normal environmental conditions and low iron. (**C**) Differences in VsHcp protein secretion of the T6SS between AJ01 and ΔVsFur. (**D**) The mRNA expression levels of AJ01 and ΔVsFur T6SS genes. The error line is the standard deviation (*n* = 3). The asterisks represent significant differences (** *p* < 0.01 by two-tailed unpaired Student’s *t*-test).

Previous studies have suggested that iron availability is closely associated with T6SS activity [21]. To determine whether iron influences T6SS function in *V. splendidus*, the expression and activity of T6SS-related genes were examined under normal and iron-deficient conditions. qPCR analysis revealed that the expression levels of the T6SS-related genes *VsHcp*, *VsClpV*, *VsVgrG*, *VsImpK*, and *VsImpJ* were significantly down-regulated under iron-limited conditions (Figure 2A). Western blot showed that the secretion of VsHcp, a hallmark protein of T6SS activity, was markedly decreased under iron-deficient conditions (Figure 2B). Consistently, a similar phenomenon was observed in the *VsFur*-knockdown condition. The expression levels of T6SS-associated genes were significantly reduced in the *VsFur*-knockdown strain, reaching only 22–53% of those in the wild-type strain (Figure 2C). In addition, the secretion of VsHcp was markedly decreased in the *VsFur*-knockdown strain (Figure 2D). The highly similar expression patterns observed under iron limitation and *VsFur* knockdown suggest that *VsFur* may participate in the iron-dependent regulation of T6SS in *V. splendidus*.

### 3.3. VsFur Directly Binds to the Promoter Region of the T6SS Gene Cluster

**Figure 3:**
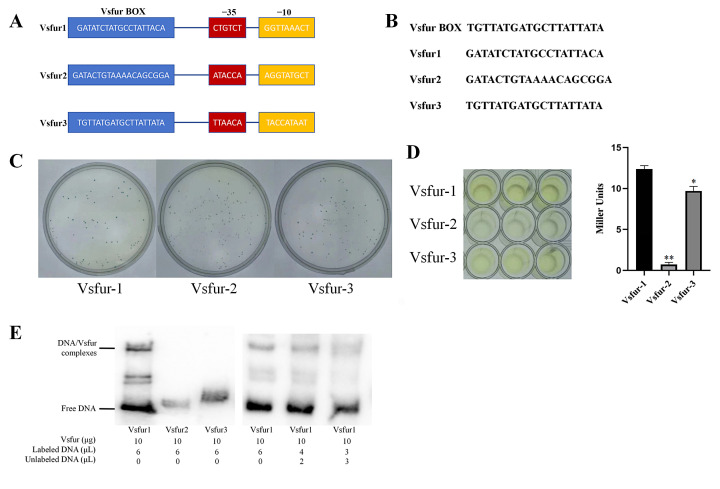
(**A**) *V. splendidus* T6SS predicts *VsFur* BOX-containing promoter regions. (**B**) Sequence comparison of the *VsFur* BOX region. (**C**) The X-gal assay detects promoter activity of strains containing promoter-recombinant vectors. (**D**) The ONPG assay detects promoter activity of strains containing the promoter-recombinant vector. The error line is the standard deviation (*n* = 3). The asterisks represent significant differences (* *p* < 0.05, ** *p* < 0.01 by one-way ANOVA). (**E**) VsFur protein was tested for binding ability by EMSA assay with labeled DNA probes and competitive binding using unlabeled probes.

Given that Fur is a global iron-responsive transcriptional regulator and T6SS expression was significantly reduced under both iron-deficient and *VsFur*-knockdown conditions, we hypothesized that *VsFur* may directly regulate T6SS transcription. To verify this hypothesis, the promoter region of the T6SS gene cluster was analyzed for potential Fur-binding sites. Three promoter regions containing conserved Fur-binding motifs were discovered and designated as Vsfur1, Vsfur2, and Vsfur3, respectively (Figure 3A,B). All three promoter regions were approximately 300 bp in length and contained typical bacterial promoter elements, including the −35 and −10 boxes, as well as putative Fur-binding sequences with more than 50% similarity to the consensus Fur box.

To evaluate their promoter activities, the three promoter fragments were cloned into the pSC11 reporter vector and analyzed using X-gal and ONPG assays. The results showed that Vsfur1 exhibited the strongest promoter activity (12.3 Miller units), whereas Vsfur2 displayed the weakest activity (0.75 Miller units). Vsfur3 showed intermediate promoter activity (9.7 Miller units) (Figure 3C,D). The result suggested that Vsfur1 may represent the major regulatory promoter within the T6SS gene cluster. Further, we performed EMSA to determine whether VsFur directly interacts with the T6SS promoter. The results showed that a clear mobility shift was observed only in the Vsfur1 probe. Moreover, the shifted band was progressively reduced by the addition of unlabeled competitor DNA and markedly weakened at a 1:1 ratio of unlabeled-to-labeled probe (Figure 3E). These results demonstrate that VsFur directly binds to the promoter region of the T6SS gene cluster, providing evidence for its direct involvement in T6SS transcriptional regulation.

### 3.4. VsFur Promotes the Secretion and Translocation of T6SS Effector Proteins

**Figure 4:**
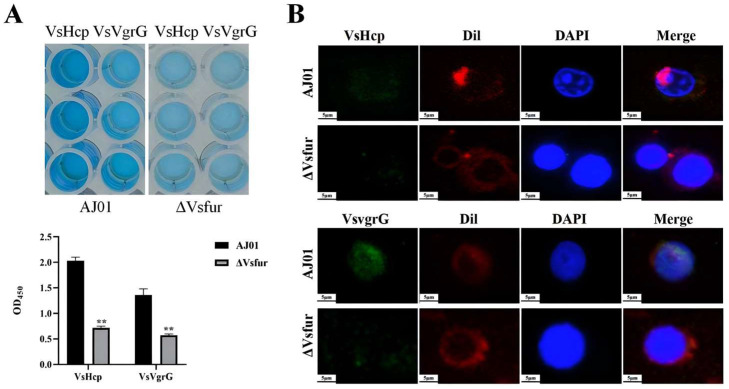
(**A**) ELISA detection of AJ01 and ΔVsFur strains for VsHcp, VsVgrG T6SS protein secretion. The error line is the standard deviation (*n* = 3). The asterisks represent significant differences (** *p* < 0.01 by two-tailed unpaired Student’s *t*-test). (**B**) Immunofluorescence experimental detection of the ΔVsFur strain for the secretion of VsHcp, VsVgrG T6SS proteins. Green signals respresent VsHcp and VsvgrG. Blue and red signals represent nucleus and cell membrane, respectively. The AJ01 as a control, scale bar = 5 μm.

To further determine whether *VsFur*-mediated regulation of T6SS transcription affects the secretion and delivery of T6SS effector proteins, the extracellular secretion and host–cell translocation of VsHcp and VsVgrG were analyzed in the wild-type strain and the *VsFur*-knockdown strain. ELISA analysis revealed that knockdown of *VsFur* significantly reduced the extracellular secretion of both VsHcp and VsVgrG. The protein levels of VsHcp and VsVgrG were decreased to approximately 1/3 of those observed in the wild-type strain (Figure 4A). Consistently, immunofluorescence analysis demonstrated that the cellular entry of T6SS effector proteins was markedly reduced in the *VsFur*-knockdown strain compared with the wild-type strain (Figure 4B). These results indicate that *VsFur* positively regulates the secretion and translocation of T6SS effector proteins in *V. splendidus*.

### 3.5. Knockdown of VsFur Gene Increased Bacterial Hemolytic Capacity and Iron Carrier Production

**Figure 5:**
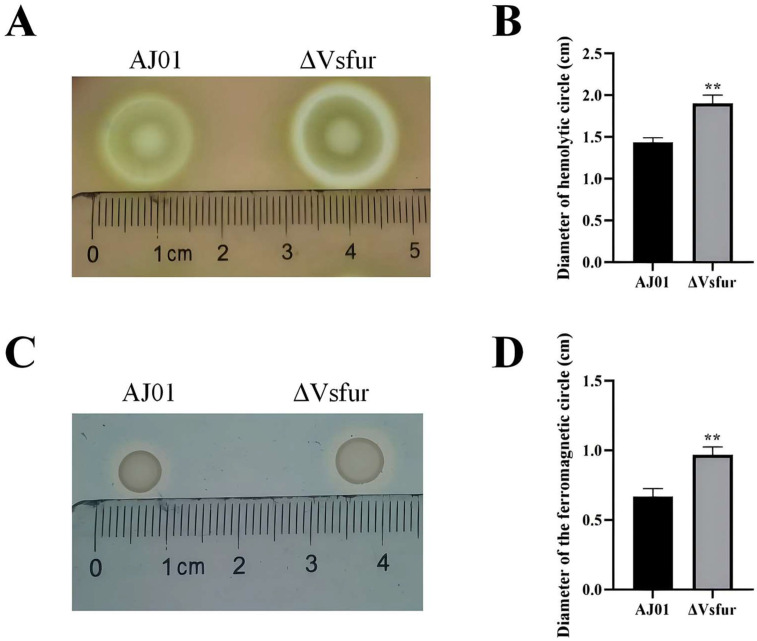
(**A**) Hemolytic circles of the two experimental strains, AJ01 and ΔVsFur. (**B**) Difference in hemolytic circle diameter between the two experimental strains, AJ01 and ΔVsFur. (**C**) Hemophilic circles of the two experimental strains, AJ01 and ΔVsFur. (**D**) Difference in the diameter of the ferrophilic circles of the two experimental strains, AJ01 and ΔVsFur. The error line is the standard deviation (*n* = 3). The asterisks represent significant differences (** *p* < 0.01 by two-tailed unpaired Student’s *t*-test).

Given that Fur is a global regulator involved in both virulence regulation and iron homeostasis, we further investigated whether *VsFur* knockdown affected hemolytic activity and siderophore production in *V. splendidus*. Compared with the wild-type strain, the *VsFur*-knockdown strain exhibited a significantly larger area of hemolytic circle (Figure 5A,B). Similarly, the siderophore halo of the *VsFur*-knockdown strain exhibited a larger area compared to the wild type (Figure 5C,D). These results indicated that *VsFur* knockdown enhanced hemolytic activity and siderophore production of *V. splendidus*. The observed alterations in multiple virulence-associated phenotypes following *VsFur* knockdown suggested that *VsFur* may regulate a broader virulence network in *V. splendidus*.

## 4. Discussion

Iron is an essential micronutrient that is required for numerous bacterial physiological processes, including metabolism, growth, and virulence [29]. However, hosts usually restrict iron availability through nutritional immunity during infection, which creates a hostile environment for invading bacteria [30]. To overcome this limitation, bacteria have evolved sophisticated iron acquisition systems and regulatory networks that coordinate iron homeostasis with pathogenicity [31]. Among these regulators, Fur serves as a central iron-responsive transcriptional regulator that coordinates iron homeostasis and virulence-associated functions in a wide range of bacterial pathogens. In the present study, we demonstrated that knockdown of *VsFur* significantly altered multiple virulence-associated phenotypes in *V. splendidus*, including bacterial aggregation and adhesion. These findings indicate that *VsFur* functions as a global regulator linking iron metabolism with bacterial pathogenicity. Similar regulatory functions have been reported in numerous bacterial pathogens, where Fur modulates biofilm formation, adhesion, motility, toxin production, and host colonization [32]. Our findings further extend these observations and highlight the importance of Fur-mediated regulation in marine pathogenic *vibrios*.

Our study systematically revealed the central role of VsFur proteins in the regulation of *V. splendidus* T6SS function by integrating iron metabolism and virulence export. We found that both iron limitation and *VsFur* knockdown significantly reduced the transcription of multiple T6SS-associated genes and suppressed Hcp secretion. The finding suggests that T6SS activity is positively regulated by iron through a Fur-dependent pathway. Similar regulatory mechanisms were also reported in other bacterial pathogens. In *Vibrio parahaemolyticus*, Fur directly regulates the balance of T6SS-1 and T6SS-2 by dynamically responding to changes in iron concentration in order to adapt to different host microenvironments [33]. Likewise, Fur coordinates iron homeostasis and virulence through interaction with the EsrB regulatory pathway controlling T3SS and T6SS expression in *Edwardsiella piscicida* [34]. Although these specific regulatory mechanisms were not investigated in the present study, they provide supporting evidence that Fur-mediated regulation of secretion systems is a conserved strategy among bacterial pathogens for coordinating iron homeostasis with virulence [35].

As a DNA-binding transcriptional regulator, Fur commonly controls gene expression through direct interaction with promoter regions containing Fur-binding motifs [36]. Consistently, promoter prediction analysis identified conserved Fur-binding sequences within the T6SS gene cluster of *V. splendidus*. EMSA assays further demonstrated that VsFur specifically binds to the Vsfur1 promoter region. These findings provide direct evidence that *VsFur* regulates T6SS at the transcriptional level. Similar mechanisms have been reported in *P. aeruginosa*, where Fur participates in the regulation of T6SS-associated genes through promoter interactions [37]. In addition, studies in *Edwardsiella tarda* have suggested that Fur may influence secretion system expression through interactions with other transcriptional regulators, including H-NS-mediated silencing pathways [38,39]. Therefore, we speculated that additional regulatory factors (epigenetic modifications or interactions with other regulatory factors) may also contribute to the Fur-dependent regulation of T6SS in *V. splendidus*.

The regulatory pathway was further supported by analyses of T6SS effector proteins. Knockdown of *VsFur* markedly reduced both extracellular secretion and host intracellular effector protein delivery of Hcp and VgrG. These findings uncovered a novel role for *VsFur* in regulating T6SS function, extending its role beyond the classical control of iron metabolism. Although the underlying mechanism remains unclear, previous studies have provided several possible explanations. For instance, Fur deficiency has been associated with impaired T6SS function through ROS-mediated oxidation of T6SS sheath proteins in *V. cholerae* [17,40]. More notably, another study reported that defective T6SS activity in the ΔVsFur mutant may also result from perturbation of quorum sensing (QS). Fur in *Vibrio fischeri* regulates the expression of the QS master protein LuxR by binding sRNA, which directly activates the transcription of T6SS gene clusters [41]. Together, these observations suggest that Fur may coordinate T6SS activity through multiple regulatory pathways, thereby linking environmental adaptation to bacterial competition and virulence. Whether these mechanisms are involved in *VsFur*-mediated regulation of T6SS in *V. splendidus* requires further investigation.

Notably, knockdown of *VsFur* reduced bacterial aggregation and adhesion but increased hemolytic activity and siderophore production. This phenotypic paradox reveals a metabolic reprogramming strategy of pathogenic bacteria under resource constraints. On the one hand, the T6SS effector protein VgrG promotes adhesion by disrupting the host cytoskeleton or membrane structure, and its insufficient secretion directly impairs the invasion efficiency. On the other hand, *VsFur* knockdown activates the expression of iron carriers and hemolysin, which releases hemoglobin iron by lysing the host cells to compensate for the iron uptake. A similar strategy is observed in *Staphylococcus aureus* under iron-restricted conditions. The bacteria hijack host iron resources by up-regulating hemolysin and iron carrier expression [42,43]. However, this compensation may exacerbate the host’s inflammatory response, ultimately limiting bacterial colonization. Therefore, bacteria may balance multiple virulence determinants to optimize environmental adaptation and host colonization.

## 5. Conclusions

**Figure 6:**
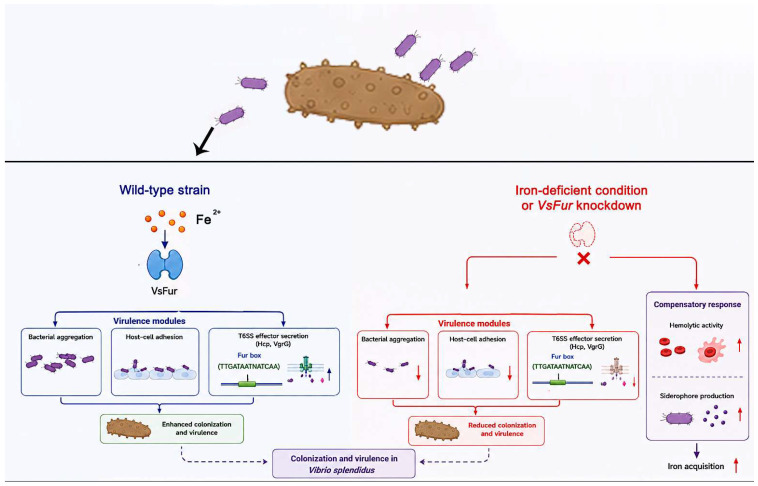
Schematic diagram of *VsFur* coordinating virulence and iron homeostasis in *V. splendidus*. Activated T6SS promotes bacterial aggregation, adhesion, and virulence. In contrast, knockdown of *VsFur* suppresses aggregation, adhesion, and T6SS activity, but increases hemolytic activity and siderophore production, indicating a compensatory response for iron acquisition. Overall, *VsFur* acts as a central regulator linking iron homeostasis to bacterial virulence in *V. splendidus*.

In conclusion, this study reveals a previously unrecognized role of *VsFur* in coordinating iron homeostasis and virulence in *V. splendidus* (Figure 6). These findings expand our understanding of iron-dependent pathogenic mechanisms in marine bacteria and provide a theoretical basis for the development of anti-virulence strategies against bacterial diseases in *A. japonicus* aquaculture. Future studies are needed to further analyze how Fur synergizes with other environmental signals (pH, temperature, community sensing) to dynamically regulate the T6SS, and to explore the potential of inhibitors targeting the pathway (iron-chelating agents or Fur antagonists) in anti-infective therapy.

## Figures and Tables

**Table 1:** The primer sequences used in this study.

Primers	Sequence (5′-3′)	Used For
Vsfur1F Vsfur1R Vsfur2F Vsfur2R Vsfur3F Vsfur3R EMSA-F EMSA-R M13-F M13-R 16s-F 16s-R qVsFur-F qVsFur-R qVsHcp-F qVsHcp-R qVsVgrG-F qVsVgrG-R q16S-F q16S-R	AGATTCAACCTGACTGTCTTCATCG GTGCTGATAGCTCAGATCGCT AATTTTATCTCTAACAAAAGGAGAAGATTC TTTACCGTTGGAGGGATGTTTT CGTAACGAGATTTTTGTGTTCGTC AAGATGTTCTGACATAAGGTTATGTCG CGACGTTTGTAAAACGACGGCCAGT TTTCACACAGGAAACAGCTATGACC ACTGGCCGTCGTTTTACA AACAGCTATGACATGA AGAGTTTGATCCTGGCTCAG GGTTACCTTGTTACGACTT CCAGACTGCCAACATATTAGTG TTGCCTTTGCAATCCCCAGT TTGAGGGTACGACACCAAAAGG ATTTCAACAGTGCGGCCTTC TTCCATCAGTATCGAAGCCAG ACTTAATGAGCCTTCAGCTGACAG GCACAAGCGGTGGAGCATGTGG CGTGTGTAGCCCTGGTCGTA	Promoter activity EMSA probe Universal primer Real-time PCR

## Data Availability

The original contributions presented in this study are included in the article. Further inquiries can be directed to the corresponding authors.

## Abbreviations

The following abbreviations are used in this manuscript:

VsFur	ferric uptake regulator
T6SS	type VI secretion system
AJ01	wild-type strain
ΔVsFur	*VsFur*-knockdown strain
